# Detritus-based assemblage responses under salinity stress conditions in a disused aquatic artificial ecosystem

**DOI:** 10.1186/2046-9063-9-22

**Published:** 2013-12-05

**Authors:** Fulvio Cerfolli, Bruno Bellisario, Corrado Battisti

**Affiliations:** 1 Department of Ecological and Biological Sciences (DEB), Ichthyogenic Experimental Marine Centre (CISMAR), Tuscia University, Borgo Le Saline, 01016, Tarquinia, VT, Italy; 2 ‘Torre Flavia’ LTER (Long Term Environmental Research) Station, Environmental Service, Provincia di Roma, via Tiburtina, 691, 00159, Rome, Italy

**Keywords:** Leaf-detritus, Macrozoobenthos, Abundance/Biomass Comparisons, Whittaker plots, Simpson index, Nestedness, Patchy environment

## Abstract

**Background:**

Despite the plethora of approaches, the sensitivity of the methods to measure the relationship between the abundance and biomass curves in stressed detritus-based ecosystems still remain to be refined. In this work, we report the comparison between biomass and abundance in a set of detritus-based macrozoobenthic assemblages located in six sampling pools with different salinity in an artificial aquatic ecosystem (disused Tarquinia Saltworks), using two diversity/dominance approaches (Abundance/Biomass Comparisons, or ABC, and Whittaker plots). We also evaluated the contribution of abundances and biomasses diversity (Simpson index) and nestedness, which measures the order by which macroinvertebrates colonized the detrital resource.

**Results:**

The outputs obtained by both ABC curves and Whittaker plots highlight two different thresholds in assemblage structure: between about 44 and 50 practical salinity unit (psu) and between 50 and 87 psu, respectively. The first threshold was due to a turnover in taxon composition between assemblages, the second threshold (evidenced by Whittaker plots) was due to a change in taxon richness (lower in pools with higher salinity: i.e. > 50 psu). Moreover, a normal-shaped pattern in diversity (Simpson index) emerged, suggestive of an intermediate disturbance effect. The nested pattern did not show significant differences when considering the density and biomass of the sampled taxa, providing similar threshold of salinity in the relative contribution of macrozoobenthos on nestedness.

**Conclusions:**

The use of detailed (ABC and Whittaker plots) and macroscopic (Simpson index and nestedness) approaches is proposed to identify thresholds in the structuring and functioning of detritus-based community of disused aquatic ecosystems: in particular, the inclusion of the parameter of biomass (scarcely utilized in community-based research) appears crucial. The responses of macrozoobenthic assemblages to the salinity stress conditions, in term of abundance and biomass, using a detritus food source (*Phragmites australis* leaves), may also highlight, by comparing macroscopic and detailed approaches, structuring and functioning patterns to consider for the management of disused artificial ecosystems.

## Introduction

The aquatic ecosystems represent a test bench to study the structures and functioning of the heterotrophic macrobenthic assemblages under stress conditions [1,2]. In the trophic structures based on autochthonous and allochthonous plant detritus inputs, the role of heterotrophy is crucial to canalize energy and organic materials [3,4]. To understand the importance of detritus-based macrozoobenthic assemblages in terms of trophic exchanges, investigations on the donor-controlled properties of detritus-based energy channels are essential [5]. It is well known that in the aquatic ecosystems, the chemical stressors (i.e. salinity, dissolved oxygen, pH) represent a driving force for the organization of the ecological community assemblages in relative species-specific abundances and biomasses [6,7]. In community ecology, large datasets on the abundance and biomass of species assemblages [8] may be analyzed using different approaches [9-11]. Among them, the Abundance/Biomass Comparisons (or ABC curves) and the diversity/dominance diagrams (or Whittaker plots) [12-14] have been largely utilized. All the diagrams obtained by these analyses make explicit the frequency ratio (or dominance) among species, either calculated on individual abundance (e.g., in diversity/dominance diagrams) or cumulatively, based on abundance and biomass at the same time (e.g., ABC curves). These representations provide an explicit information on the structure of species assemblages (e.g., diversity and evenness), but they also allow to assess the stress level that might functionally affect the organisms: for instance, in the Whittaker plots, more elevated abundance curves represent the less diverse and more stressed assemblages, while in the ABC curves the comparison between the biomass and abundance curves is used to make inferences on the level of disturbance affecting the taxonomic assemblages [15]. Therefore, knowing the abundance and biomass of the taxonomic assemblages, the Simpson index is profitably used to verify the presence of a normal-shaped pattern in diversity [11]. Concerning other approaches, it is useful to analyse specific mechanisms involved in the structuring processes of ecological communities, since they may exert a strong influence on both the stability and functioning of ecosystems [16], partly reflecting the extent to which interspecific competition is involved in communities composition [17]. One possible approach to investigate patterns and mechanisms involved in community composition, to be proactively coupled with the approaches set out above, is the calculation of nestedness [18], which refers to the ‘linkage order’ observed between elements of different sets (i.e. species/inlands [19] and plant and pollinator [18]). Macroscopically, the nestedness in habitat/resource colonization occurs when the species present in species-poor sites are proper subsets of the assemblages found in species-rich sites [20]. A perfect nested structure occurs when all species-poor sites are proper subsets of the assemblages found in richer species sites [21]. However, absence of nestedness does not always mean absence of structural pattern, as many other assemblages can be observed in the structuring process of ecological communities (e.g., gradients and compartments) [22,23]. Therefore, the correct evaluation of the mechanisms involved in such structures may provide useful information on the stability and functioning of detritus-based communities [24].

The sensitivity of the methods to weigh the relationship between the biomass and abundance curves in stressed detritus-based ecosystems remains to be explored in detail [3,25] in particular in disused aquatic ecosystems. Indeed, the ABC method and Whittaker plots have been recently applied to some vertebrate and invertebrate assemblages [26-29], but not to detritus-based macrozoobenthic assemblages, a significant component of heterotrophic food webs [3].

Recent works emphasized that stress conditions disrupt the abundance/biomass relationships (in ABC curves) and the evenness (in Whittaker plots) in species assemblages [15] and affects the diversity and the biomass of communities of primary producers in streams [30]. Conversely, diversity indices (Shannon’s and Simpson’s), abundance and biomass, during breakdown have been largely adopted in detritus-based communities [31].

Moreover, useful topological properties of network assemblage (e.g. nestedness) have been used to measure the role of salinity in the structuring and functioning of artificial aquatic ecosystems [32] with emphasis on predicting the mechanisms behind the ecological patterns in macrozoobenthic assemblages. In this work, we compared biomass with abundance in a set of detritus-based macrozoobenthic assemblages sampling on *Phragmites australis* (Cav.) Trin. ex Steud., leaf detritus in six sites with different salinity, located in the disused Tarquinia Saltworks, an artificial aquatic ecosystem of central Italy. We applied ABC curves and Whittaker plots to compare the cumulative abundance and biomass data (ABC curves) and the ranking in relative abundance (Whittaker plots) obtained from these macrozoobenthic assemblages to evaluate their responses under salinity stress conditions. We extended the Abundance/Biomass Comparison method using the Simpson index, a macroscopic approach, to investigate the responses of donor-controlled communities [33] due to salinity variation in terms of diversity.

To test the sensitivity of the structuring and functioning of the macrozoobenthic assemblages, we also measured nestedness, a well-known structural characteristic of the complex networks [18,21] with a linkage to the functional attributes of the systems [32].

## Methods

### Study area

**Figure 1:**
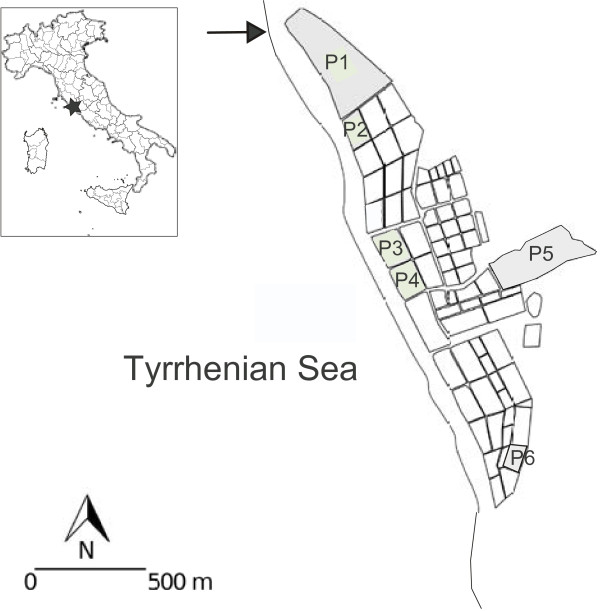
**Spatial location of the six sampling pools in the Natural Reserve of Tarquinia Saltworks.** Black arrow indicates the main point of water refill between the sea and the study area, while the numbered surfaces highlighted in grey shows the sampling sites.

The study area is the aquatic ecosystem of disused Tarquinia Saltworks, a patchy environment (central Italy, 42°12′ N, 11°43′ E), composed by a series of about 100 pools whose connection is ensured by a surrounding drainage system. The exchange of waters is provided by a single connection with the sea located north of the area (Figure 1). Isolation and hydrological connectivity give rise to a wide salinity gradient [7], spanning from hypohaline (mean annual salinity 8.515 psu or gL^-1^) to hyperhaline waters (mean annual salinity 115.000 psu or gL^-1^), (Table 1).

**Table 1:** Main chemical–physical parameters measured in the six sampling pools

**POOL**	**P5**	**P1**	**P2**	**P3**	**P4**	**P6**
**Salinity (psu)**	8.515 (±4.292)	44.769 (±4.746)	50.531 (±5.126)	87.000 (±10.083)	100.154 (±12.548)	115.000 (±29.958)
**[O**_**2**_**] mgL**^**-1**^	10.03 (±1.644)	5.765 (±2.543)	6.068 (±2.703)	5.505 (±1.568)	5.865 (±1.216)	5.536 (±1.779)
**pH**	8.953 (±0.566)	8.112 (±0.243)	8.104 (±0.252)	8.067 (±0.296)	8.134 (±0.236)	8.034 (±0.187)

Values are expressed as mean ± SD.

### Field and laboratory methods

The spatial characteristics of the pools within the study area (e.g. isolation and connectivity), give rise to a wide environmental gradient, showing a spatial pattern in the variability of the salinity levels, pH and dissolved oxygen concentration [32].

Six sampling sites (pools) were randomly selected, covering the maximal range of salinity variation from hypohaline (mean annual salinity 8.515 psu) to hyperhaline waters (mean annual salinity more than 115.000 psu), (Table 1), to perform a macrozoobenthic colonization experiment of *Phragmites australis* (Cav.) Trin. ex Steud. leaf detritus, naturally present in the area, under different salinity stress conditions. To measure the relationship between cumulative abundance and biomass frequencies and salinity, we placed in each pool 48 protected (mesh size: 5 × 5 mm) and 48 unprotected (mesh size: 10 × 10 mm) leaf packs. We measured, on a monthly sampling with r = 4 replicates: i) the dry weight of leaf detritus in both protected and unprotected leaf packs, after storing at 60°C for at least 72 h (leaf pack initial weight: 2.000 ± 0.004 g dry mass) [7]; ii) the number of colonizing taxa (first column in Table 2); iii) the number of individuals for each taxon (expressed as means with n = 4 replicates); iv) the dry biomass of individuals for each taxon (expressed as means with n = 4 replicates), after storing at 60°C for at least 72 h, and the determination of the ash free dry weight (AFDW) after ignition in a muffle furnace at 500°C for 6 h [34]. The loss upon oxidation is referred to as AFDW. AFDW, obtained by subtracting the ash content (>60% of total dry mass) was considered to provide a better comparison with other macroinvertebrate taxa than dry weight [35].

**Table 2:** Detritus-based assemblages in the six pools studied, ordered by increasing salinity

**POOL**	**P5**		**P1**		**P2**		**P3**		**P4**		**P6**	
**Taxon**	**FrN**	**FrB**	**FrN**	**FrB**	**FrN**	**FrB**	**FrN**	**FrB**	**FrN**	**FrB**	**FrN**	**FrB**
*Chironomus* sp. (larvae)	0.167	0.014	0.239	0.014	0.536	0.371	0.757	0.180	0.928	0.586	0.994	0.980
*Gammarus aequicauda*	0	0	0.268	0.023	0.034	0.008	0	0	0	0	0	0
*Perinereis culltrifera*	0	0	0.058	0.062	0.003	0.010	0	0	0	0	0	0
*Nereis diversicolor*	0	0	0.262	0.490	0.034	0.175	0	0	0	0	0	0
*Hydrobia acuta* (complex)	0	0	0.087	0.072	0.140	0.312	0.216	0.728	0.003	0.028	0	0
*Cerastoderma glaucum*	0	0	0.003	0.013	0.003	0.042	0.004	0.070	0.005	0.225	0	0
(other) Coleoptera (larvae)	0.014	0.002	0.026	0.002	0	0	0	0	0	0	0	0
*Cerithium vulgatum*	0	0	0.052	0.324	0	0	0	0	0	0	0	0
Gordiidae	0	0	0.003	0	0	0	0	0	0	0	0	0
*Spio decorates*	0	0	0	0	0.007	0.004	0	0	0	0	0	0
(other) Diptera (larvae)	0.007	0.001	0	0	0.003	0.001	0	0	0	0	0	0
*Monocorophium insidiosum*	0	0	0	0	0.225	0.063	0	0	0	0	0	0
*Idotea balthica*	0	0	0	0	0.014	0.012	0	0	0	0	0	0
*Haliplus* sp.	0.530	0.182	0	0	0	0	0.023	0.022	0.064	0.162	0.004	0.015
*Micronecta* sp	0.035	0.008	0	0	0	0	0	0	0	0	0	0
Anisoptera (nymphae)	0.220	0.705	0	0	0	0	0	0	0	0	0	0
*Acilius* sp (larvae)	0.021	0.086	0	0	0	0	0	0	0	0	0	0
*Hydrophilus* sp	0.007	0.002	0	0	0	0	0	0	0	0	0.002	0.005
Taxonomic units	8		9		10		4		4		3	
Abundance (total N ind)	287		343		293		518		643		519	
Biomass (grams, AFDW)		2.679		4.727		1.483		1.739		0.815		0.421

Abundance frequency (frN) and biomass frequency (frB) for each taxon, number of taxonomic units, total number of macrozoobenthic individuals (N) and total macrozoobenthic biomass (B) are reported.

### Data analysis

For each taxon in each sampling site, we obtained values of abundance (N) and biomass (B), and for both we calculated their relative (frN and frB) and cumulative frequency (Table 2). We then ranked the taxa from the most to the least important based on either cumulative abundance or biomass along the x-axis in a Cartesian space in order to obtain two curves for these parameters (ABC curves).

Whittaker plots were obtained building a taxon rank/relative frequency diagrams, and utilizing the data set (taxon/frequency) applied to values in frequency on abundance (log-transformed to improve normality and for literature comparison [11]) of the six detritus-based assemblages studied. A first-degree equation was calculated by fit analysis for each detritus-based assemblage, including all taxa. The equation is Fr*A* = *b*^*ar*^, where Fr*A* is the relative frequency (on the abundance) of each taxon in each pool; *r* is the rank of each taxon in the pool assemblage; *a* is the angular coefficient (negative) of the regression line, indicating the mean decrease of the relative frequency of the taxon with increase of the taxon rank (slope of the line); *b* is a coefficient (intercept value) that reflects the trend value of the first dominant taxon of the pool assemblage represented in the regression line in equation.

For each regression line we obtained the coefficient of determination (R^2^) as an estimate of the variance explained [36]. For each taxon assemblage we also calculated the Simpson diversity indexes as D = 1 – Σ fr^2^, both on the abundance and biomass frequencies (D_N_ and D_B_). The index provides a good estimate of diversity with a relatively small sample size, being less sensitive to taxon richness and capturing the variance of the taxon abundance (or biomass) distribution [11]. To compare the abundance and biomass frequency distributions between pools we performed the Kolmogorov-Smirnov 2-sample test. Probability level (*p*) was set at 0.05. We investigated taxon assemblages as whole units where relative abundance frequency of the taxon is a value aimed to obtain regression lines in the Whittaker plots. Therefore, the results did not allow a discussion on possible implications on assembly rules among taxa determined by interspecific competition, predation or disturbance at species level.

We evaluated also the nested pattern of macrozoobenthic assemblage on *P. australis* leaf detritus in the six sampling sites by taking into account the weights of the association between taxa and sites. Although nestedness originally relies on the presence/absence of a particular association in the association matrices [19], recent advances suggest the role of weighting the intensity of such association for a thorough understanding of the mechanism involved [37].

Two bipartite networks were then created to study the role of abundances and biomasses on nestedness. A bipartite network is defined by two distinct sets of nodes (in our case macroinvertebrates and resource/sites), where links may occur only between the nodes of different sets but not within nodes of the same set. To account for the relative contribution of species abundance and biomass on nestedness, we used a weighted version of nestedness based on the Manhattan distance (WINE, Weighted-Interaction Nestedness Estimator) [38]. Although WINE has been criticized due to its tendency to overestimate nestedness for matrices with no co-occurrence among species and/or for matrices with sites of identical richness [39], some authors suggested its capability to measure the relative contribution of species to nestedness when dealing with abundance data [40]. Weighted nestedness was measured with the WINE function implemented in the “bipartite” package of R [41]. WINE takes into account the weight or intensity of each entry (e.g. the abundances and biomasses of sampled macroinvertebrates). The nestedness score of the data matrix is normalized by comparing it to the average score of equivalent random matrices and to the score of the maximal nestedness matrix to obtain the weighted-interaction nestedness estimator [37]:

(1) $$\eta_{w}=\left(d^{w}-d_{\text{rnd}}\right)/\left(d_{\text{max}}-d_{\text{rnd}}\right)$$

where d^w^ is the mean weighted distance of all its non-zero elements, d_rnd_ is the average value of 1,000 replication random matrix and d_max_ the distance of the completely packed matrix. η_w_ varies between 0, when the score of the original data matrix is close to the average score of the equivalent random matrices, and 1, as it gets closer to the nestedness of the maximal nestedness matrix. To assess the significance of nestedness, a Z-score measuring the difference between d^w^ and d_rnd_ is calculated. Z values below −1.65 or above 1.65 indicate approximate statistical significance at the 5% error level (one-tailed test). WINE also calculates a weighted-interaction distance (d_ij_^w^), which estimates nestedness taking into account the number of events in the links, in our case the abundance and biomass of macrozoobenthos sampled on leaf detritus. A distance-based permutational multivariate analysis of variance was then performed [42] to test the influence of salinity on the contribution of abundance and biomass on nestedness. The ‘adonis’ function in package ‘vegan’, implemented in the R software environment [43], was used for partitioning distance matrices among sources of variation. Although similar to the classic PERMANOVA, the function ‘adonis’ is more robust, as it can accept both categorical and continuous variables. We used average salinity as fixed factor, to test for its influence on the relative contribution of abundance and biomass in different assemblages. The Bray-Curtis resemblance matrices were constructed, and significance was tested by performing 999 permutations of both abundances and estimated biomasses within each group, which were defined following the salinity gradient.

We finally compared the ranking of sites derived from nestedness (measured on both the abundance and densities) with the ranking yielded by the angular coefficient given by the best fitting model of the Whittaker plots, to look for a correspondence between nestedness and pattern of species distribution.

## Results

Fluctuations in the level of pH and dissolved oxygen concentration slightly affected the environmental conditions within the pools, as showed by the PCA ordination that explained 83.41% of total variance, mainly attributable to the variation of salinity within the pools (*r* = 0.96).

A total of 2,603 individuals (11.864 AFDW grams) were collected, belonging to S = 18 macrozoobenthic taxa in P = 6 pools.

**Figure 2:**
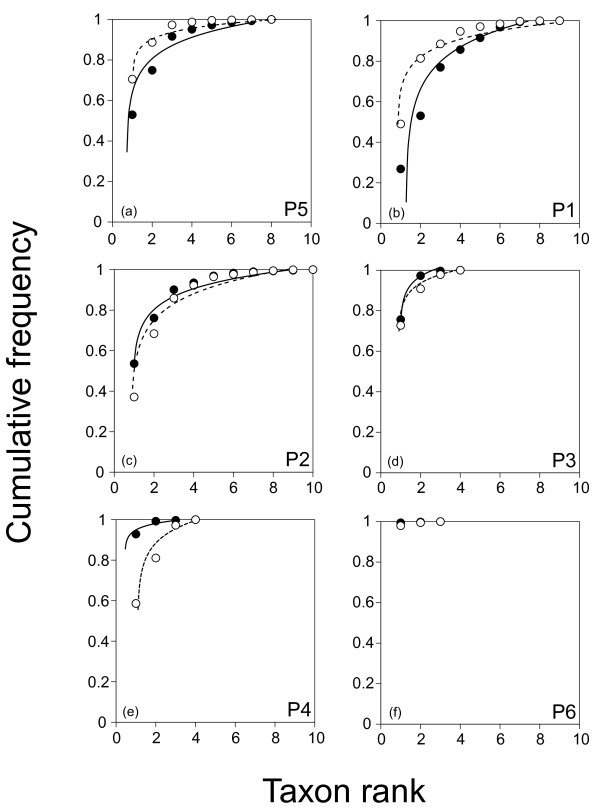
**Abundance/Biomass Comparisons (ABC curves) for the six sampling pools.** Values of cumulative frequency on abundance (black circles; continuous line) and biomass (white circles; dashed line) along a taxon ranking (x-axis) are reported. Figures 2**abc** refer to pools with salinity < 50 psu, while Figures 2**def** to pools with values of salinity > 50 psu.

In the Abundance/Biomass Comparison method, we detected a change in relative patterns of cumulative abundance and biomass. In particular, between about 44 e 50 psu, we observed a change in the relative position of abundance and biomass curves. Until 44 psu, the biomass curves cumulate before the abundance curves, and then the abundance curves cumulate before the biomass curves (Figure 2). The frequency distribution for abundance and biomass was significantly different in all the pools, except in the pool with elevated salinity level (Table 3).

**Table 3:** **Comparison between abundance and biomass frequencies by Kolmogorov-Smirnov 2-sample test (Z with *p*) for macrozoobenthic assemblages in the six sampling pools**

**POOL**	**P5**	**P1**	**P2**	**P3**	**P4**	**P6**
**Z (*p*)**	2 (<0.01)	1.741 (<0.01)	2.415 (< 0.01)	1.414 (< 0.05)	1.414 (<0.05)	1.225 (= 0.1)
**Fr** ***A***	235.8e^-0.66×^	334e^-0.61×^	194.28e^-0.60×^	2933.5e^-1.81×^	2703e^-1.97×^	5214.5e^-3.12×^
***R***^**2**^	0.95	0.89	0.95	0.99	0.93	0.83
***a***	122	181.4	107	480	377	230.26

The relative equations (Fr*A*), the coefficients of determination (*R*^*2*^) and the angular coefficients (*a*) are also listed for each pool.

In Whittaker plots, we observed two sets of regression lines well fitting the assemblage values (i.e. all with a high coefficient of determination > 0.80). A first set of regression lines, characterized by a low slope (ranging between −0.60 and −0.66), including the three detritus-based assemblages living in pools with a salinity lower than 50 psu; a second set, characterized by a higher slope (ranging between – 1.81 and −3.12), including the three assemblages living in pools with a salinity higher than 50 psu (Figure 2abc).

**Figure 3:**
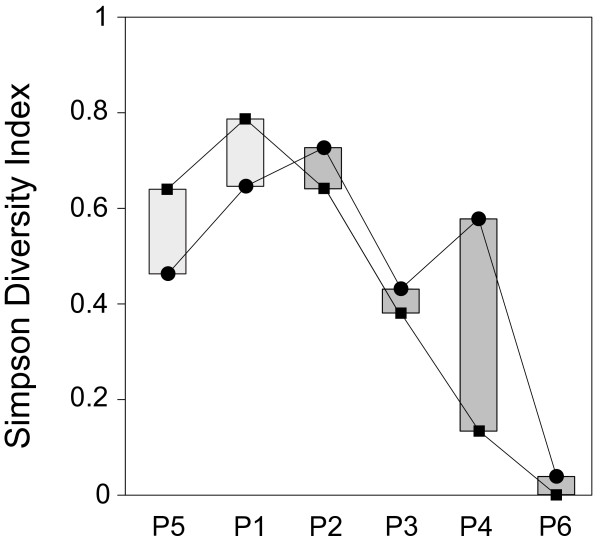
**Values of the Simpson diversity index calculated both on abundance (DN, black squares) and biomass (DB, black circles) frequencies for the six sampling pools.** Vertical bars are the differences between diversity indexes (DN-DB), with light grey bars representing positive values (DN > DB) and dark grey bars negative values (DN < DB).

Comparing the Simpson indexes calculated on abundance (N) and biomass (B) frequencies, two patterns apparently normal-shaped but shifting among them were observed: D_N_ peaks in pool n. 1 (psu = 44.769) and D_B_ peaks in pool n. 2 (psu = 50.531) (Figure 3).

The results obtained by both ABC curves and Whittaker plots highlight two different thresholds: between 44–50 and 50–87 psu, respectively. The first threshold (evidenced by the ABC curves) seems due to a turnover in taxon composition between assemblages (from taxon with higher biomass and less abundance to taxon with lower biomass and higher abundance); the second threshold (evidenced by Whittaker plots) is due to a change in taxon richness (lower in pools with higher salinity: i.e. > 50 psu).

A significant nested structure was found for the association between macrozoobenthos and the detrital resource (measure of the availability of *P. australis* detritus following the variation patterns of decomposition [6]) in different pools either when considering the abundance (*η*_w_ = 0.265, *Z* = 2.142, *p* = 0.02) or the biomass of sampled taxa (*η*_w_ = 0.412, *Z* = 3.414, *p* < 0.001). The contribution of macrozoobenthos to nestedness varied between pools following the salinity gradient (*F* > 75 and *p* = 0.01 for both abundances and biomasses), highlighting a threshold in the salinity values able to affect the nested assemblage of macroinvertebrates on leaf detritus.

The ranking comparison between nestedness and pattern of species distribution was also highly significant (Spearman’s rank coefficient test: *r*_s_ = 0.889, *p* < 0.01).

## Discussion

Following the Abundance/Biomass Comparison model, a change in relative location of the cumulative biomass vs. abundance curves implies a change in the level of disturbance affecting specific assemblages. The theory on the ABC curves assumes that when the abundance curves cumulate before (i.e. are higher), an assemblage may be stressed by a disturbance [11]. In our detritus-based assemblages, this disturbance could be due to an increasing level of salinity, starting from values higher than 44 psu. As for the influence of salinity, the adoption of ordination analyses is useful in providing information on the relative importance of chemical factors on the macrozoobenthic communities. The ABC curves emphasize the different ecological role that the abundance and biomass parameters play at the taxon assemblage level. In particular, abundance curves indicate a relative distribution of the spatial niche and dominance of the taxonomic unities, while biomass curves indicate the relative distribution of the energy flow in the assemblage, according to the trophic resources used by taxa [10]. The Abundance/Biomass Comparisons are based on the assumption that in disturbed habitats, small-sized and generalist taxa (i.e., with low body weight, low trophic level and/or r-selected) tend to increase in their abundance. As a consequence, in these ecosystems the abundance curves approach an asymptote before the biomass curves. On the contrary, in undisturbed habitats the opposite pattern could be observed, with the biomass curves cumulating before the abundance curves. This may suggest that a higher number of large-sized taxa of high trophic level occur in a more complex and diverse assemblage. Following this approach, early cumulating abundance curves may indicate that the resources are used by few dominant (i.e., more abundant) taxa with a broad spatial niche (i.e., generalist), while early cumulating biomass curves may indicate that the individuals with high body weight (and taxa with a high biomass) largely occur in the undisturbed assemblage typical of stable ecosystems [11]. Therefore, when abundance and biomass curves are compared, we may obtain information on the level of anthropogenic or natural disturbance that affects a taxon assemblage by alternating relative dominance patterns among large and specialized taxa *versus* small and generalist ones.

Following this general model, the observed pattern suggests that the detritus-based macrozoobenthic assemblages are stressed by a different level of disturbance starting from a salinity level of 50 psu. Between about 44 and 50 psu, we observed a pattern where the relationship between abundance and biomass of macrozoobenthic assemblages allows the presence of taxa with many individuals but low biomass.

Differently, Whittaker plots emphasized a threshold between 50 and 87 psu of salinity, especially due to a strong reduction in taxon richness and an over-dominance of the remnant generalist taxa with a high relative abundance. Indeed, when salinity increases at very high level, only a low number of taxa (i.e. larvae of *Chironomus* sp.) survive, so demographically increasing their dominance [7,32].

Therefore, the information on change in both taxon composition and taxon richness is intercepted from two diversity/dominance approaches (ABC curves and Whittaker plots). When analyzing assemblages along gradients, the use of different approaches at assemblage level may help to identify different thresholds: in particular, the parameter of biomass (scarcely utilized in community-based research) appears crucial to describe the relationship between abundance and biomass [35].

Finally, we observed a normal-shaped pattern in diversity (Simpson index), suggesting the presence of an intermediate disturbance effect [44,45]. This effect predicts that diversity will be the highest in assemblages with intermediate levels of disturbances. In this case, taxon richness is maximized because of the coexistence between salinity-tolerant generalist taxa and other more sensitive taxa. These assemblages differ in term of abundance and biomass, thereby inducing a shift (between 44 and 50 psu) in the higher values of two diversity indexes.

Concerning the metrics of network analysis, the contribution of macrozoobenthic assemblages to nestedness varied among pools following the salinity gradient (*F* > 75 and *p* = 0.01 for both abundances and biomasses), highlighting the presence of a threshold in the salinity values able to influence the nested assemblages on leaf detritus.

The disused Tarquinia Saltworks showed, in the early stages, an intense process of macrozoobenthic colonization [46]. The single pools, characterized by different residual salinity, thus become, macroscopically, a patchy environment with distinct trophic structures [32]. Along with the structuring of the green trophic structures, due to the colonization of aquatic submersed vegetation (i.e. *Ruppia cirrhosa* (Petag.) Grande) and microalgae, other trophic dynamics are established upon the input of allochthonous plant debris such as that resulting from riparian plants (i.e. *Phragmites australis)*. The detritus-based macrozoobenthic assemblages constitute the primary consumers in the brown trophic food webs of Mediterranean coastal lagoons [47]. However, the long-term disuse of large aquatic artificial systems leads to a loss of structural and functional heterogeneity resulting in a homogenization of trophic structures and a decrease of the values of biological diversity.

Our work suggests that the heterogeneity of the macrozoobenthic assemblages of patchy aquatic ecosystems into disuse, a result due to the processes in the short to medium term ecological colonization, is maintained over the long term through targeted contrast homogenization and abundance and biomass monitoring activities on macrozoobenthic assemblages, independently from the scale [48]. The highest values of taxon diversity fall between 44 and 50 psu, at intermediate values in salinity. As a first approximation, the analysis of shifts in ABC curves enables to understand when, in these artificial patchy environments, the action is needed to maintain in confined areas high levels of taxon diversity and diversification of trophic structures [49], with taxon of higher biomass. Above 44 psu in salinity, the taxa tends to decrease their biomass and a taxon turnover occurs qualitatively changing the assemblage composition. Above 50 psu in salinity, these systems tend to abruptly decrease their taxon richness due to extreme conditions favouring only widely diffused taxa.

The results of the analysis of both macroscopic (diversity indexes and network metrics) and detailed patterns (ABC and Whittaker plots), based on abundance and biomass analysis, suggest that the sampling of abundances and biomasses of the macrozoobenthic assemblages is useful to increase the predictive capacity to test the sensitivity of the responses of detritus-based communities under salinity stress.

The exploitation of more ecological techniques to unravel the abundance and biomass relationships is necessary to refine the management criteria of aquatic environments with high heterogeneity, especially disused artificial ecosystems as they can represent a good model for patchy ecosystem management [50].

These results highlight also the relevance to adopt more kinds of community-based approaches to probe the patterns of macrozoobenthic assemblages, before adopting a patchy ecosystem management criterion.

## Competing interests

The authors declare that they have no competing interests.

## Authors’ contributions

FC and CB contributed to the conceptual development of the work. FC wrote the ms and carried out the statistical analyses with CB and BB. All the authors read and approved the final version of the manuscript.
